# A Specimen-Based Comparative MicroCT–FEA Analysis of Vertebral Trabecular Bone Microarchitecture and Mechanical Response in Two South American Cervids: The Patagonian Huemul (*Hippocamelus bisulcus*) and the Southern Pudu (*Pudu puda*)

**DOI:** 10.3390/biology15090722

**Published:** 2026-05-02

**Authors:** Danae Tapia, Álvaro González, Fernando Vidal, Paulo Salinas

**Affiliations:** 1 Laboratory of Animal & Experimental Morphology, Institute of Biology, Faculty of Sciences, Pontificia Universidad Católica de Valparaíso, Valparaíso 2340000, Chile; danae.tapia.m@mail.pucv.cl; 2 MSc Biological Sciences Program, Institute of Biology, Faculty of Sciences, Pontificia Universidad Católica de Valparaíso, Valparaíso 2340000, Chile; 3 Laboratorio de Investigación en Mecánica Computacional, Escuela de Ingeniería Mecánica, Pontificia Universidad Católica de Valparaíso, Quilpué 2430000, Chile; alvaro.gonzalez.o@pucv.cl; 4 Fauna Andina, Wildlife Conservation and Management Center, Villarrica 4930000, Chile; fauna.andina@gmail.com; 5 International Union for Conservation of Nature (IUCN), Deer Specialist Group, Apple Valley, MN 55124, USA; 6 International Union for Conservation of Nature (IUCN), Conservation Planning Specialist Group, Apple Valley, MN 55124, USA

**Keywords:** trabecular, bone, vertebral column, finite element analysis, micro-computed tomography, deer

## Abstract

The Patagonian huemul (*Hippocamelus bisulcus*) and the Southern pudu (*Pudu puda*) are native South American cervids that differ in body size, ecology, and conservation status. However, quantitative evidence linking vertebral trabecular microarchitecture with biomechanical behavior in these species remains scarce. This study aimed to comparatively characterize vertebral trabecular bone structure and its mechanical response using an integrative, non-destructive approach. Vertebral bodies from cervical, thoracic, and lumbar regions were analyzed using high-resolution micro-computed tomography to quantify structural parameters, followed by finite element analysis to estimate deformation and von Mises stress under standardized axial compression. Both specimens exhibited consistent regional variation, with cervical vertebrae showing lower density and organization, and thoracic–lumbar vertebrae displaying denser trabecular networks. The Southern pudu specimen appeared to present a more homogeneous microarchitecture and a relatively uniform mechanical response along the vertebral column. In contrast, the Patagonian huemul specimen tended to show greater structural heterogeneity, with apparently higher deformation and stress values, particularly in the cervical region. These findings suggest that trabecular organization may contribute to the differences in vertebral mechanical behavior observed between the analyzed specimens. This study provides a preliminary comparative baseline for understanding skeletal adaptation and structural vulnerability in South American cervid species. This exploratory analysis is based on single specimens per species and should be interpreted as preliminary evidence rather than population-level inference.

## Simple Summary

The Patagonian huemul and the Southern pudu are two native deer species from South America that differ markedly in body size and ecological context. Understanding how their bones are structured and how they respond to mechanical loads can provide valuable information for both biology and conservation. In this study, we analyzed the internal structure of the vertebrae, focusing on the trabecular bone, which plays a key role in supporting loads. We found that both species showed differences along the vertebral column, with the thoracic and lumbar regions having a denser and more organized structure, while the cervical region was more porous. When comparing species, the Southern pudu exhibited a more uniform structure and mechanical response, whereas the Patagonian huemul showed greater variability and higher levels of deformation and internal stress, particularly in the neck region. These findings suggest that differences in internal bone organization may influence how the vertebral column responds to mechanical demands. This study provides a quantitative reference that may be useful for future research on skeletal adaptation, bone diseases, and conservation strategies in native South American cervid species.

## 1. Introduction

Trabecular bone tissue is a dynamic component of the skeletal system whose three-dimensional (3D) microarchitecture undergoes continuous remodeling in response to mechanical demands, in accordance with Wolff’s law [1,2,3]. In mammals, this microstructural organization varies as a function of body size, lifestyle, and locomotor requirements, following allometric scaling patterns across species [4,5,6]. The vertebral column represents a particularly relevant model for investigating these adaptations, as vertebral bodies are subjected to axial and transverse loads that generate systematic regional variations in trabecular microarchitecture along the cervical, thoracic, and lumbar segments [7,8,9].

In South American cervids, osteological studies have primarily focused on the skull, limbs, and antlers [10,11,12,13,14,15,16,17,18], whereas the axial skeleton has received comparatively limited attention. When the vertebral column of cervids has been examined, studies have predominantly approached it as a biomedical surrogate model of the human spine, focusing on morphometric comparisons of lumbar vertebrae rather than on the functional and ecological implications of vertebral trabecular organization [19,20,21,22]. Furthermore, the few comparative analyses of vertebral cancellous bone microarchitecture available in mammals have been conducted primarily in laboratory rodents, domesticated ungulates, or human cadaveric material [23,24], leaving native wild cervids—and South American species in particular—largely uncharacterized at the microstructural level. Consequently, a clear knowledge gap persists regarding regional variation in the 3D microarchitecture of vertebral bodies in native South American cervids. The mechanical implications of these structural differences under controlled loading conditions remain poorly understood [25]. This gap is particularly significant given that trabecular microarchitecture is not only a determinant of vertebral mechanical competence, but also a potential indicator of skeletal pathology, nutritional status, and habitat-related loading history in wild populations [26,27]. To date, no study has integrated vertebral trabecular microstructural analysis with finite element analysis (FEA) in these taxa, incorporating regional comparisons along the vertebral column.

From a comparative functional morphology perspective, linking trabecular microarchitecture, mechanical response, and ecological context provides a preliminary framework for interpreting skeletal adaptations in wild species, where differences in body size, habitat, and loading regime may be reflected in divergent trabecular configurations. In this context, the Patagonian huemul (*Hippocamelus bisulcus*) and the Southern pudu (*Pudu puda*), two South American cervids that differ markedly in body size, ecology, and locomotor demands, constitute high-value comparative models. These species provide an opportunity to explore how such variables are reflected in vertebral trabecular microstructural organization and mechanical response. The Patagonian huemul, classified as “*Endangered*” on the International Union for Conservation of Nature Red List [28], has an approximate body mass of 70–90 kg and inhabits rugged Andean environments [28,29]. In contrast, the Southern pudu—one of the smallest deer species worldwide (7–10 kg)—inhabits temperate rainforest ecosystems in Southern Chile and Argentina and is categorized as Near Threatened [30,31]. Both species face significant anthropogenic and environmental pressures, and in the Patagonian huemul, a high prevalence of osteopathologies associated, among other factors, with deficiencies in micronutrients essential for skeletal development has been documented [26]. Despite their ecological and conservation relevance, available information on vertebral trabecular bone microarchitecture in these species remains limited. These contrasting biological profiles—differing markedly in body size, ecological context, locomotor demands, and skeletal health status—make these two species a compelling comparative pair for addressing the identified knowledge gap regarding regional vertebral trabecular microarchitecture and its mechanical implications in native South American cervids.

Micro-computed tomography (microCT) has become the reference non-destructive tool for quantifying bone microarchitecture, enabling high-resolution 3D imaging and precise measurement of structural parameters such as connectivity and anisotropy [32,33,34]. Its integration with finite element analysis (FEA) extends this characterization toward a functional dimension, linking trabecular microarchitectural descriptors with estimates of mechanical response under controlled loading conditions—an approach particularly well suited for comparative studies in threatened species with restricted specimen availability, as it enables the identification of relative stress and strain patterns without compromising specimen integrity [35].

On the basis of these considerations, the present study was conceived as a comparative and integrative analysis of vertebral trabecular bone tissue and its mechanical response in two South American cervids of high conservation value. The results may also contribute an anatomical–functional line of evidence to the ongoing debate regarding the natural habitat of the Patagonian huemul, particularly concerning its historical association with forested versus steppe environments [36,37], though this interpretation must be considered preliminary given the exploratory design of the study. We explored whether *H. bisulcus* and *P. puda* differ in their regional vertebral trabecular microarchitecture and associated mechanical response—with particular attention to potential differences in stress magnitude and distribution along the cervical, thoracic, and lumbar segments. Accordingly, this study aimed to comparatively characterize the 3D trabecular microarchitecture and simulated mechanical response of cervical, thoracic, and lumbar vertebral bodies in both species using an integrative microCT–FEA approach. This analysis establishes a preliminary quantitative baseline for these native South American cervids. Given the limited number of available specimens, the analysis was conducted under an exploratory and comparative framework; therefore, the results should not be interpreted as population-level trends, but rather as descriptive contrasts providing preliminary evidence on trabecular structural organization and mechanical response.

## 2. Materials and Methods

### 2.1. Biological Material and Specimen Selection

**Figure 1:**
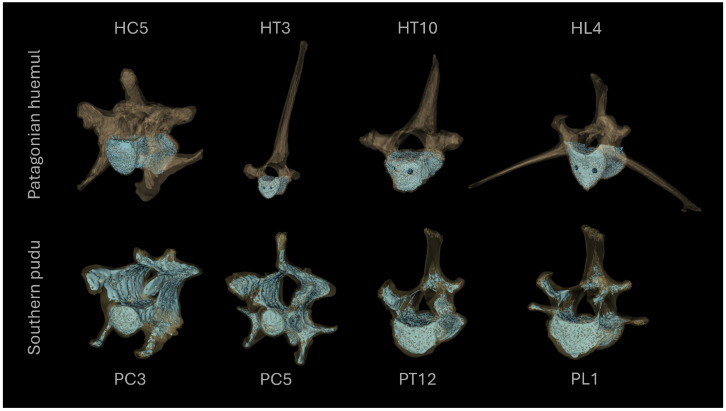
Three-dimensional reconstructions of cervical, thoracic, and lumbar vertebrae from the Patagonian huemul (*Hippocamelus bisulcus*) and the Southern pudu (*Pudu puda*) used in this study. The reconstructions correspond to the cervical (HC5), cranial thoracic (HT3), caudal thoracic (HT10), and lumbar (HL4) vertebrae of the Patagonian huemul, and the cervical (PC3 and PC5), caudal thoracic (PT12) and lumbar (PL1) vertebrae of the Southern pudu.

The study was conducted using vertebral columns from a Patagonian huemul (*Hippocamelus bisulcus*) and a Southern pudu (*Pudu puda*) (Table 1). For each species, four vertebrae were selected to represent distinct functional regions of the vertebral column, including cervical, thoracic, and lumbar segments (Figure 1). The specific vertebrae analyzed were as follows: in the Patagonian huemul, a cervical vertebra C5 (HC5), a cranial thoracic vertebra T3 (HT3), a caudal thoracic vertebra T10 (HT10), and a lumbar vertebra L4 (HL4); in the Southern pudu, two cervical vertebrae C3 and C5 (PC3 and PC5, respectively), one caudal thoracic vertebra T12 (PT12), and one lumbar vertebra L1 (PL1). This selection strategy was designed to capture well-defined morphological and structural contrasts along the cranio–caudal axis, enabling intra- and interspecific comparisons of trabecular bone architecture and its associated mechanical response. The selection of vertebral levels was constrained by specimen availability and preservation condition, precluding the use of strictly homologous elements between species; accordingly, the sampling strategy prioritized functionally comparable regions rather than exact anatomical equivalents.

Due to the high conservation status of both species and the limited access to osteological material, this study was conducted on a single specimen per species. This is therefore framed as an exploratory, specimen-based comparative analysis and does not aim to support population-level inference, consistent with previous microCT-based finite element studies conducted under restricted sample conditions [23,35]. It should be noted that the specimens differ in sex (male huemul vs. female pudu) and age (2 vs. 3 years), representing potential confounding factors that could not be controlled in the present analysis and should be considered in the interpretation of the results. The post-mortem condition of the specimens was assumed not to alter the relative trabecular architecture captured by microCT and therefore not to affect the comparative mechanical outcomes of the study. All specimens were processed under homogeneous criteria of image acquisition, segmentation, volume definition, and numerical discretization, ensuring direct comparability between vertebral regions and species.

### 2.2. MicroCT Acquisition and Image Processing

Trabecular bone tissue from the vertebral bodies was analyzed using high-resolution microCT, following established procedures for quantitative trabecular bone assessment [32,33,34]. Scans were acquired using a Bruker Skyscan 1278 system (Bruker, Kontich, Belgium) with the following parameters: 59kV, 692μA, voxel size of 51.489μm, and an exposure time of 23ms. Image reconstruction, segmentation, and quantitative processing were performed using CT Analyser software (CTAn v. 1.18.4.0, Bruker microCT). Binary segmentation was carried out using a global thresholding approach, with a threshold range of [50–255] grayscale units selected to isolate the trabecular phase from background and soft tissue signal, based on visual inspection of the grayscale histogram and verified against representative cross-sectional slices for each specimen. The selected voxel resolution ensured adequate representation of the trabecular network while maintaining compatibility with subsequent three-dimensional reconstruction and finite element discretization. Three-dimensional visualizations and reconstructions were generated using 3D Slicer (v. 5.6.2) and CTVox (v. 3.3.1, Bruker microCT) for qualitative inspection of trabecular morphology and verification of segmentation consistency.

### 2.3. Definition of Volume of Interest (VOI)

**Figure 2:**
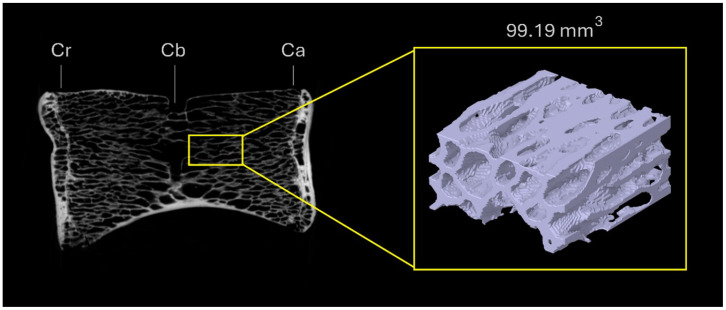
Definition and location of the volume of interest (VOI; $99.19\;{\mathrm{mm}}^{3}$) within the vertebral body. The VOI was positioned adjacent to the basivertebral canal and extended caudally, avoiding cortical bone and endplate regions. Cr: cranial end; Cb: basivertebral canal; Ca: caudal end.

For each vertebra, a standardized volume of interest (VOI) of $198.40\;{\mathrm{mm}}^{3}$ (5.72×2.99×11.28mm) was defined within the central region of the vertebral body to quantify trabecular microstructural parameters. The VOI was positioned adjacent to the basivertebral canal and extended cranially and caudally while avoiding cortical bone and endplate regions to ensure that only trabecular tissue was included in the analysis. Subsequently, the VOI was reduced to a fixed volume of $99.19\;{\mathrm{mm}}^{3}$ (5.72×2.99×5.61mm) per vertebra, corresponding to the caudal half of the originally sampled volume (Figure 2). The caudal half was selected because it consistently provided the most homogeneous trabecular region within the vertebral body, minimizing the influence of the basivertebral canal and cranial endplate irregularities on microstructural quantification. No formal evaluation of alternative VOI configurations was performed; the selection was based on anatomical criteria and established precedents in microCT-based trabecular bone analysis [34,35]. The VOI was defined to function as a representative volume element (RVE) of the trabecular microarchitecture for each vertebra, enabling consistent biomechanical comparisons across vertebral regions and species. Its final size and location were determined by balancing geometric complexity, biological representativeness, and numerical feasibility, particularly in relation to finite element modeling. For each VOI, a three-dimensional surface model preserving the original trabecular architecture was generated and exported in STL format for subsequent numerical analysis.

It should be noted that the selected VOI configuration was not validated against alternative positions or geometries; consequently, quantitative microstructural outcomes may be influenced by this choice, and results should be interpreted in the context of the specific anatomical region analyzed.

### 2.4. Finite Element Modeling

**Figure 3:**
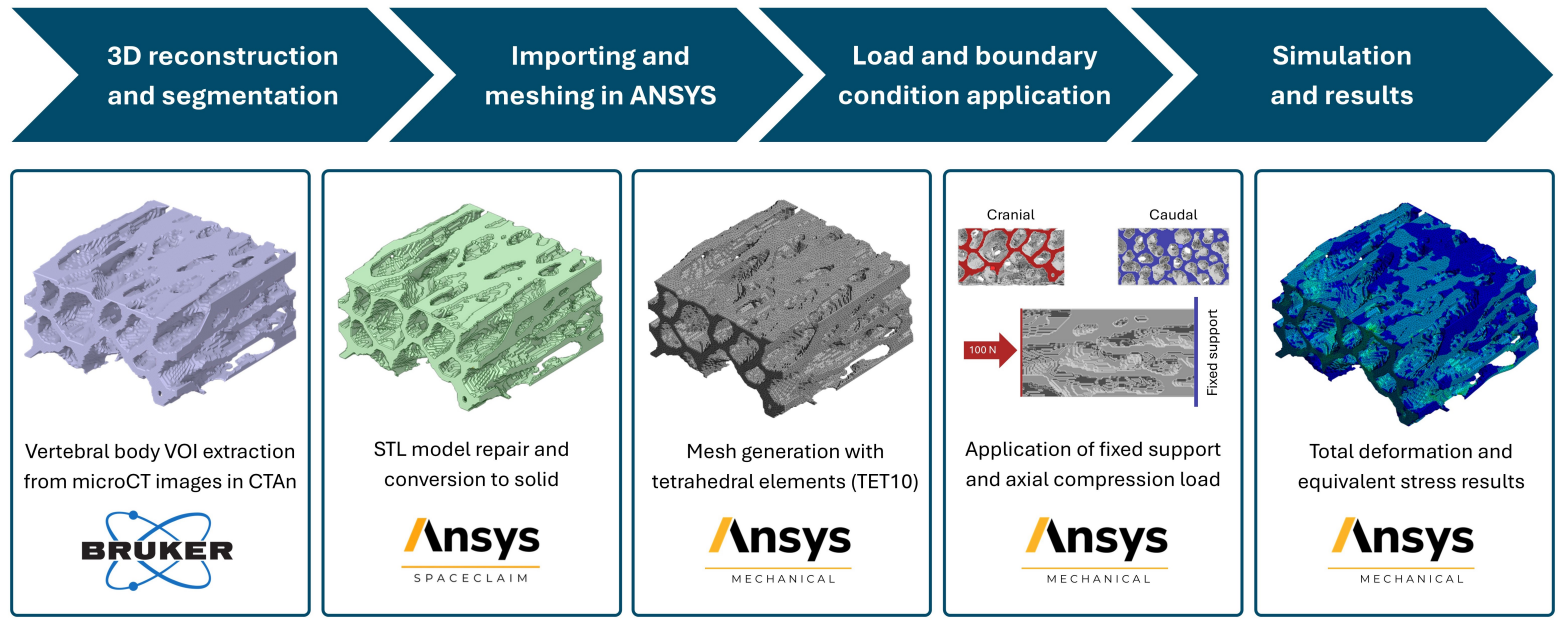
Workflow of the finite element modeling approach implemented for the biomechanical analysis of vertebral trabecular bone.

The finite element modeling workflow implemented in this study is schematically summarized in Figure 3. The methodology comprised a sequence of well-defined stages, starting with three-dimensional reconstruction and segmentation of the trabecular bone volume of interest from microCT data, followed by geometric preparation, mesh generation, and numerical discretization within the ANSYS environment. Subsequently, material properties, boundary conditions, and loading protocols were consistently applied to all models to ensure direct comparability. Finally, numerical simulations were performed to obtain mechanical response variables, including stress and deformation fields. Each stage of the workflow was designed to preserve the essential features of the trabecular architecture while maintaining numerical consistency and computational feasibility. The individual steps of this modeling strategy are described in detail in the following subsections.

#### 2.4.1. Geometric Reconstruction and Mesh Generation

The STL models derived from microCT data were imported into ANSYS Workbench (v. 2024 R2, Research License; ANSYS Inc., Canonsburg, PA, USA). Geometric preprocessing was performed in the SpaceClaim module, where segmentation-related imperfections were corrected. Trabeculae not connected to the main load-bearing network were removed in order to improve numerical stability and ensure mechanical continuity of the models, avoiding non-physical stress concentrations associated with isolated elements. Disconnected trabeculae were defined as those with no shared nodes with the principal load-bearing network, identified through connectivity analysis in the SpaceClaim module. The proportion of removed elements was minor in all models, representing less than 0.6% of the total element count per geometry, and did not alter the primary trabecular architecture of the VOI. The processed geometries were converted into solid bodies and discretized in the Mechanical module using second-order tetrahedral elements (SOLID187, TET10), which are well suited for representing complex trabecular geometries. A surface-based solid reconstruction approach was adopted instead of voxel-based meshing to reduce computational cost while preserving the essential features of the trabecular architecture relevant to comparative analysis.

#### 2.4.2. Material Assumptions and Constitutive Model

Trabecular bone tissue was modeled as a linear elastic, isotropic, and homogeneous material, with a Young’s modulus of 18 GPa and a Poisson’s ratio of 0.3, based on previously reported tissue-level properties [38]. Although trabecular bone exhibits anisotropy and heterogeneity at the apparent level, this simplified constitutive model is widely used in comparative microCT-based finite element studies. Its adoption allowed the mechanical response to be predominantly governed by differences in trabecular architecture rather than by spatial variations in material properties. It should be explicitly acknowledged that the isotropic and homogeneous assumption simplifies the biological reality of trabecular bone tissue and may have influenced interspecific comparisons, particularly given the microarchitectural differences observed between species. Specifically, such assumptions may have masked potential interspecific differences in tissue-level material properties, including variations in mineralization, collagen organization, or compositional heterogeneity, that could independently modulate mechanical response beyond what was captured by architectural parameters alone.

#### 2.4.3. Boundary Conditions and Loading Protocol

**Figure 4:**
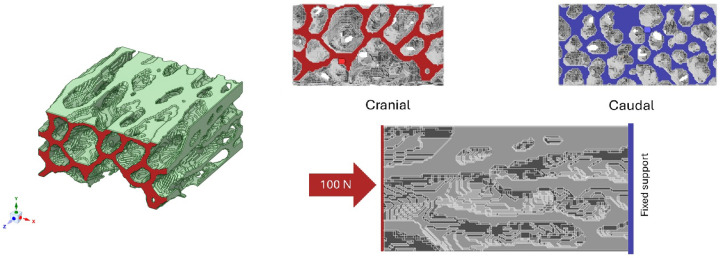
Schematic representation of the boundary conditions used in the finite element models, including a fixed support applied to the caudal surface (blue) and a uniformly distributed axial compressive load of 100 N applied to the cranial surface (red).

Standardized boundary conditions were applied to all models to ensure direct comparability. A fixed support condition was imposed on the caudal surface of the VOI, constraining all translational degrees of freedom. A compressive axial load of 100N was applied uniformly to the cranial surface and directed toward the geometric center of the analyzed volume (Figure 4). The applied load magnitude was selected in agreement with previous finite element studies investigating vertebral trabecular bone mechanics [39,40]. The applied load of 100 N was not physiological and was used solely to provide a consistent and standardized mechanical stimulus across all specimens and vertebral regions, enabling comparative assessment of relative mechanical responses rather than absolute biomechanical predictions.

#### 2.4.4. Mesh Convergence and Numerical Consistency

**Figure 5:**
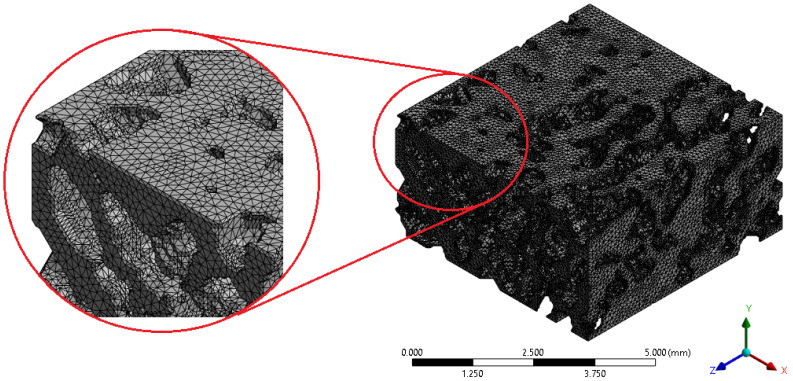
Example of the tetrahedral mesh generated, including a detailed view of the trabecular network discretization.

Mesh sensitivity was evaluated by progressively refining the element size from 1.0mm down to 0.05mm. While the average von Mises stress within the VOI showed minimal variation across the tested range, indicating early stabilization of global response metrics, mesh quality improved significantly with refinement. The finest mesh (0.05mm) consistently provided higher element quality and closely matched the microCT voxel resolution (approximately 51μm), ensuring consistency between image acquisition and numerical discretization while preserving trabecular geometry. Accordingly, this element size was adopted for all eight analyzed geometries to ensure numerical consistency and enable direct comparison between specimens. Figure 5 illustrates an example of the tetrahedral mesh generated, highlighting the accurate geometric representation of the trabecular network achieved with the selected discretization. Detailed results of the mesh convergence analysis, including stress convergence trends, mesh quality evolution, and mesh statistics, are provided in the Supplementary Material (Supplementary Material S3).

#### 2.4.5. Mechanical Variables and Data Analysis

The mechanical response of each model was evaluated in terms of the equivalent von Mises stress (MPa) and total deformation (mm). Von Mises stress was employed as a scalar indicator to facilitate visualization and comparison of stress concentration patterns within the trabecular network, while total deformation provided a measure of overall deformability under compressive loading. For each vertebra, the minimum, maximum, and average values of both variables were extracted. Spatial distributions were visualized using continuous color maps ranging from blue (minimum values) to red (maximum values), enabling qualitative assessment of load transfer pathways and regions of stress concentration. Given the exploratory nature of the study and the limited number of specimens, no inferential statistical analyses were performed. Data interpretation was therefore restricted to qualitative and relative comparisons between vertebral regions and between the analyzed specimens.

## 3. Results

### 3.1. Trabecular Microarchitecture of the Vertebral Body in the Patagonian Huemul

The quantified microstructural parameters of vertebral trabecular bone in the Patagonian huemul are summarized in Table 2. The cervical vertebra 5 (HC5) exhibited the lowest values of relative bone volume and bone surface density (BV/TV = 22.73%; BS/TV = 3.24 1/mm), but showed the highest surface-to-volume ratio (BS/BV = 14.27 1/mm). In contrast, the thoracic vertebrae (HT3 and HT10) appeared to display more connected trabecular networks (Tb.Pf = 1.29 and −0.19 1/mm, respectively) and a tendency toward greater prevalence of plate-like trabeculae (SMI = 0.60 and −0.12), whereas HC5 tended to show the lowest connectivity (Tb.Pf = 3.43 1/mm) and a predominance of rod-like trabeculae (SMI = 1.44). Trabecular thickness remained relatively constant (Tb.Th = 0.24 ± 0.08 mm), with a localized increase observed in HT10 (0.30 ± 0.09 mm). The lowest trabecular separation was recorded in the lumbar vertebra 4 (HL4; Tb.Sp = 0.58 ± 0.20 mm), whereas HT10 presented the apparently greatest separation (0.67 ± 0.30 mm). Trabecular number appeared to increase progressively from the cervical region (Tb.N = 0.90 1/mm) toward the lumbar region (Tb.N = 1.21 1/mm), with a similar fractal dimension among vertebrae (FD ≈2.6). Total porosity was highest in HC5 (77.27%) and lowest in HT10 (64.53%), while HT3 and HL4 showed intermediate and comparable values (≈73%). Regarding anisotropy, HC5 exhibited a low degree (DA = 0.30), in contrast to the remaining vertebrae, which appeared highly anisotropic (DA ≈0.80).

### 3.2. Trabecular Microarchitecture of the Vertebral Body in the Southern Pudu

The microstructural parameters of vertebral trabecular bone in the Southern pudu are presented in Table 3. The caudal thoracic vertebra (PT12) exhibited the highest relative trabecular bone volume (BV/TV = 36.47%) and, together with the cervical vertebra PC3, the highest bone surface density values (BS/TV = 3.80 1/mm). The greatest surface-to-volume ratio (BS/BV = 13.13 1/mm) was observed in the cervical vertebra PC5. The lumbar vertebra (PL1) appeared to show the most connected trabecular network (Tb.Pf = −0.31 1/mm) and a tendency toward predominance of plate-like trabeculae (SMI = −0.18), whereas PC3 exhibited the lowest connectivity (Tb.Pf = 2.07 1/mm). Trabecular thickness appeared relatively homogeneous (Tb.Th = 0.30 ± 0.11 mm), with slightly lower values in PC5 (0.25 ± 0.10 mm). PC3 showed the lowest trabecular separation and number (Tb.Sp = 0.58 ± 0.27 mm; Tb.N = 1.06 1/mm), whereas PC5 exhibited the apparently greatest separation (Tb.Sp = 0.69 ± 0.34 mm) and PT12 the highest trabecular number (Tb.N = 1.31 1/mm). The highest porosity was recorded in PC5 (77.45%) and the lowest in PT12 (63.53%). In terms of anisotropy, PC5 displayed low values (DA = 0.35), while the remaining vertebrae tended to exhibit moderate to high anisotropy (DA = 0.48–0.70).

### 3.3. Interspecific Microstructural Comparison of Vertebral Trabecular Bone

The comparative analysis revealed descriptive differences in the organization of vertebral trabecular bone between the analyzed specimens. In the Patagonian huemul, bone volume (BV) ranged from 45.11 mm^3^ in HC5 to 70.37 mm^3^ in HT10, with BV/TV values varying between 22.73% and 35.47%. In the Southern pudu, BV ranged from 56.46 mm^3^ (PC5) to 72.35 mm^3^ (PT12), with apparently more uniform BV/TV values (28.46–35.13%). Trabecular thickness in the Patagonian huemul varied between 0.25 ± 0.09 mm (HC5) and 0.30 ± 0.09 mm (HT10), whereas in the Southern pudu it remained between 0.28 ± 0.08 mm and 0.30 ± 0.11 mm. Bone surface (BS) appeared slightly greater in the Southern pudu (maximum 754.67 mm^2^ in PT12) than in the Patagonian huemul (maximum 736.82 mm^2^ in HL4). The Southern pudu appeared to exhibit greater overall trabecular connectivity, whereas trabecular number was comparable between specimens, showing a cranio-caudal increase in both cases. Trabecular separation tended to be greater in the Southern pudu specimen, while the Patagonian huemul appeared to display more closely spaced trabeculae in the lumbar region. Total porosity reached maximum values in HC5 of the Patagonian huemul (77.27%) and minimum values in PT12 of the Southern pudu (63.53%), with similar fractal dimensions between specimens. In both specimens, anisotropy appeared lower in the cervical vertebrae and tended to increase progressively toward the thoracic and lumbar regions.

### 3.4. Mechanical Response from Finite Element Analysis (FEA)

#### 3.4.1. Mesh Quality and Convergence

The mesh convergence analysis confirmed that the global mechanical response of the trabecular models stabilizes rapidly as the mesh is refined. The average von Mises stress exhibited only minor variations across the range of tested element sizes, indicating that the global stress response is relatively insensitive to further mesh refinement once a moderate discretization density is achieved. For all Patagonian huemul vertebrae, the variation of the average stress remained within a narrow range when the element size decreased from 1mm to 0.05mm, demonstrating that the numerical solution reached a stable regime. Despite this early stabilization of the global stress metric, mesh refinement resulted in a clear improvement in element quality. Coarser meshes produced lower average element quality due to geometric distortion of tetrahedral elements within slender and highly curved trabecular regions. In contrast, the refined mesh (0.05mm) yielded consistently higher element quality values, exceeding 80% for all vertebral geometries. Importantly, the same discretization criteria and convergence strategy were subsequently applied to the Southern pudu vertebral models, ensuring that comparisons between the analyzed specimens were performed under identical numerical conditions. Detailed results of the mesh convergence analysis, including stress trends, mesh quality evolution, and mesh statistics, are provided in the Supplementary Material (Supplementary Material S3).

#### 3.4.2. Global Mechanical Response

The global mechanical response of the trabecular bone VOIs was evaluated in terms of total deformation and equivalent von Mises stress under standardized axial compression. The results obtained for the Patagonian huemul and the Southern pudu are summarized in Table 4 and Table 5, respectively. For the Patagonian huemul, the average von Mises stress appeared relatively consistent across the vertebral regions, ranging from 15.95 MPa in HT10 to 21.22 MPa in HT3. The cervical vertebra (HC5) and lumbar vertebra (HL4) presented intermediate values of 20.53 MPa and 18.18 MPa, respectively. Maximum stress values showed notably higher variability, reaching 818.44 MPa in HC5 and 731.84 MPa in HT3. These peak values should not be interpreted as physiological *in vivo* stresses nor as failure-level thresholds, as they reflect localized numerical concentrations under a standardized, non-physiological compressive load applied solely for comparative purposes. Accordingly, mean stress values are considered the primary comparative metric throughout this analysis. These elevated peak values are consistent with well-documented numerical behavior in microCT-based finite element models of trabecular bone, where highly localized stress concentrations arise at trabecular junctions and thin struts due to geometric singularities in the complex trabecular network [23,35]. Accordingly, mean stress values are emphasized as the primary comparative metric throughout this analysis, as they are less sensitive to such local numerical artifacts. A similar trend was observed for total deformation, with mean values remaining within a narrow range and maximum values showing more pronounced regional differences, particularly in HL4 ($7.52\times 10^{-2}$ mm). For the Southern pudu, average von Mises stress values tended to be lower than those observed in the Patagonian huemul specimen, ranging from 14.26 MPa in PC3 to 19.22 MPa in PC5. Maximum stress values appeared more uniform compared to the Patagonian huemul, varying between 346.05 MPa and 447.62 MPa across all regions. Mean deformation values were also apparently lower, ranging from $2.80\times 10^{-3}$ mm to $4.49\times 10^{-3}$ mm, with maximum values appearing more homogeneous across vertebral regions.

#### 3.4.3. Stress Distribution Patterns

**Figure 6:**
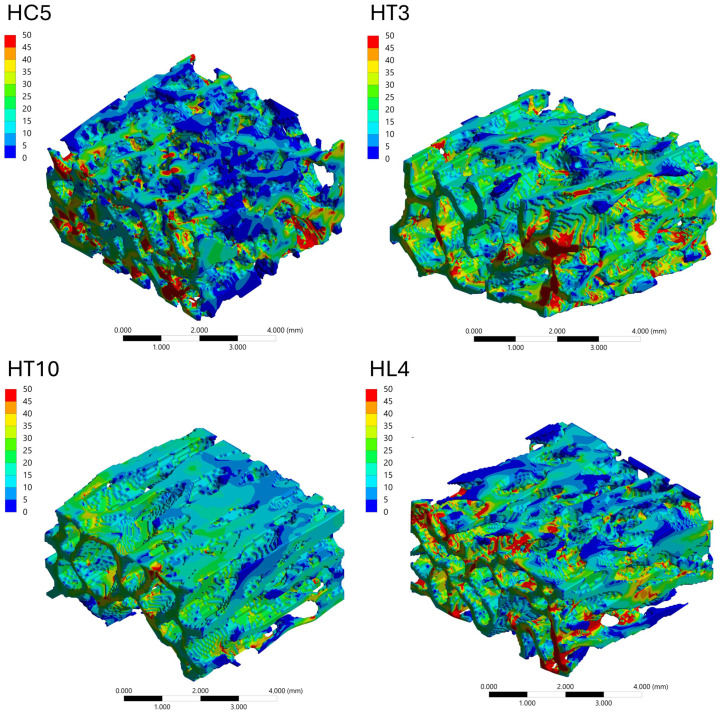
Spatial distribution of von Mises equivalent stress (MPa) in vertebral trabecular bone tissue of the Patagonian huemul (*Hippocamelus bisulcus*). Color maps correspond to the cervical (HC5), cranial thoracic (HT3), caudal thoracic (HT10), and lumbar (HL4) vertebrae. A species-specific color scale is applied to maximize visualization of intraspecific regional patterns; for direct interspecific comparison of stress magnitudes, see Figure 10. The color scale represents stress magnitude in MPa, with red tones indicating maximum values and blue tones indicating minimum values.

**Figure 7:**
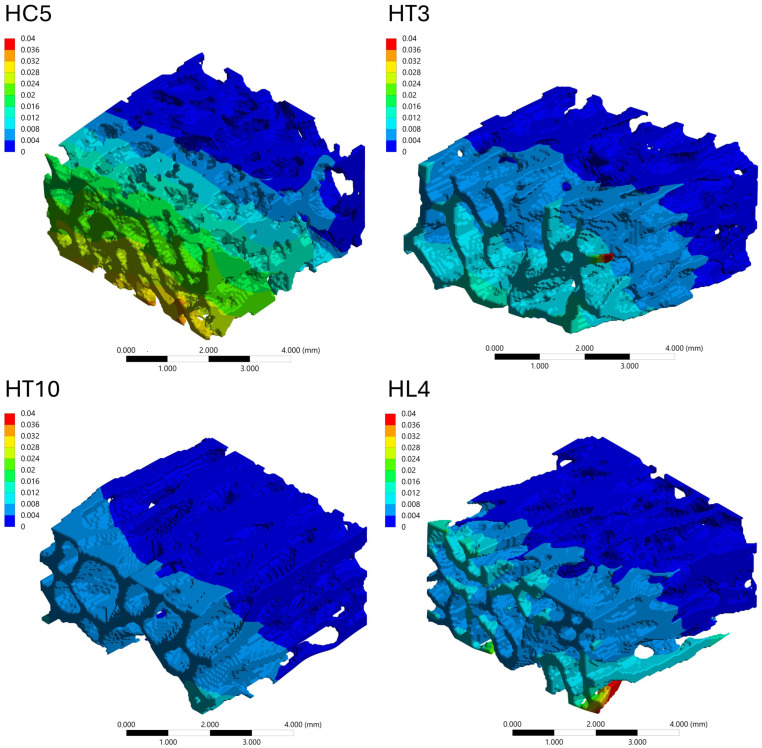
Spatial distribution of total deformation (mm) in vertebral trabecular bone tissue of the Patagonian huemul (*Hippocamelus bisulcus*). Color maps correspond to the cervical (HC5), cranial thoracic (HT3), caudal thoracic (HT10), and lumbar (HL4) vertebrae. A species-specific color scale is applied to maximize visualization of intraspecific regional deformation patterns. Deformation values reflect the response to a standardized axial compressive load of 100 N applied solely for comparative purposes and do not represent physiological in vivo conditions. The color scale represents deformation magnitude in mm, with red tones indicating maximum values and blue tones indicating minimum values.

**Figure 8:**
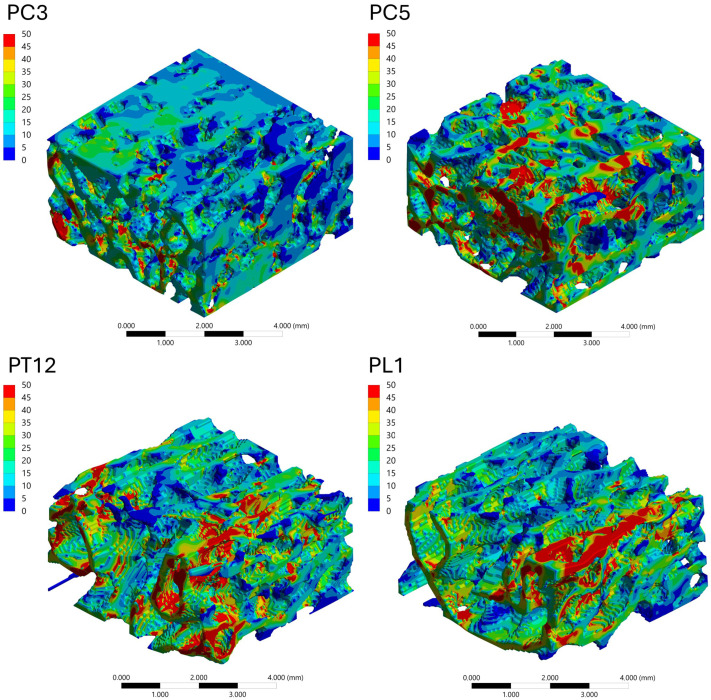
Spatial distribution of von Mises equivalent stress (MPa) in vertebral trabecular bone tissue of the Southern pudu (*Pudu puda*). Color maps correspond to the cervical (PC3 and PC5), thoracic (PT12), and lumbar (PL1) vertebrae. A species-specific color scale is applied to maximize visualization of intraspecific regional patterns; for direct interspecific comparison of stress magnitudes, see Figure 10. The color scale represents stress magnitude in MPa, with red tones indicating maximum values and blue tones indicating minimum values.

**Figure 9:**
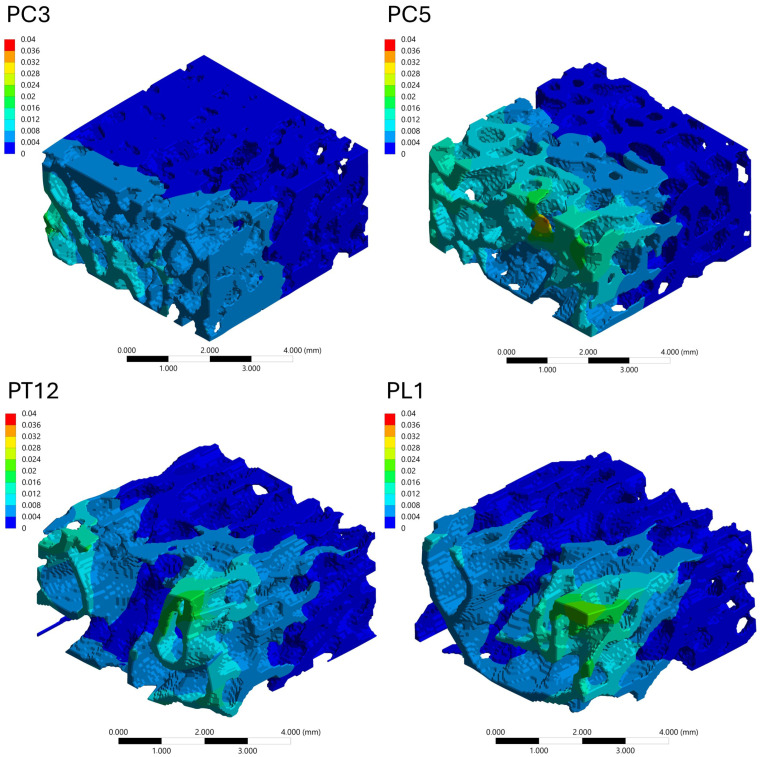
Spatial distribution of total deformation (mm) in vertebral trabecular bone tissue of the Southern pudu (*Pudu puda*). Color maps correspond to the cervical (PC3 and PC5), thoracic (PT12), and lumbar (PL1) vertebrae. A species-specific color scale is applied to maximize visualization of intraspecific regional deformation patterns. Deformation values reflect the response to a standardized axial compressive load of 100 N applied solely for comparative purposes and do not represent physiological in vivo conditions. The color scale represents deformation magnitude in mm, with red tones indicating maximum values and blue tones indicating minimum values.

The spatial distribution of mechanical response within the trabecular bone VOIs was analyzed through von Mises stress and total deformation fields, as illustrated in Figure 6, Figure 7, Figure 8 and Figure 9. Independent but internally consistent color scales were used for stress and deformation due to their different orders of magnitude. Deformation maps exhibited smooth and continuous gradients, whereas von Mises stress distributions showed highly localized patterns. For the Patagonian huemul (Figure 6), stress concentrations were predominantly localized at trabecular junctions and along vertically aligned struts. The cervical (HC5) and cranial thoracic (HT3) vertebrae tended to exhibit a more heterogeneous stress distribution, with multiple regions of elevated stress (red zones) dispersed throughout the structure. In contrast, HT10 appeared to show a more uniform stress field with fewer high-stress concentrations. The lumbar vertebra (HL4) presented distinct localized stress concentrations, particularly near trabecular clusters. For the Southern pudu (Figure 8), stress distributions appeared generally more homogeneous across all vertebral regions. The cervical vertebrae (PC3 and PC5) exhibited moderate stress heterogeneity, while the thoracic (PT12) and lumbar (PL1) regions tended to show more continuous stress fields with fewer pronounced peaks.

**Figure 10:**
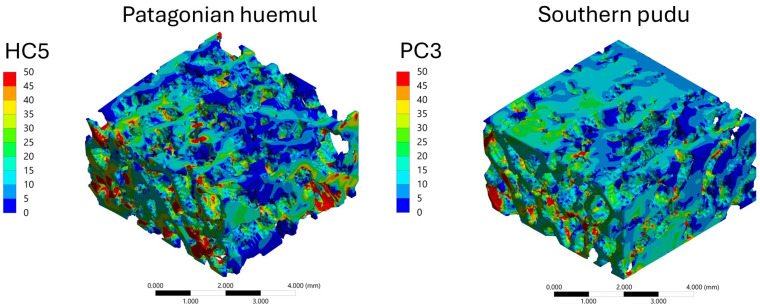

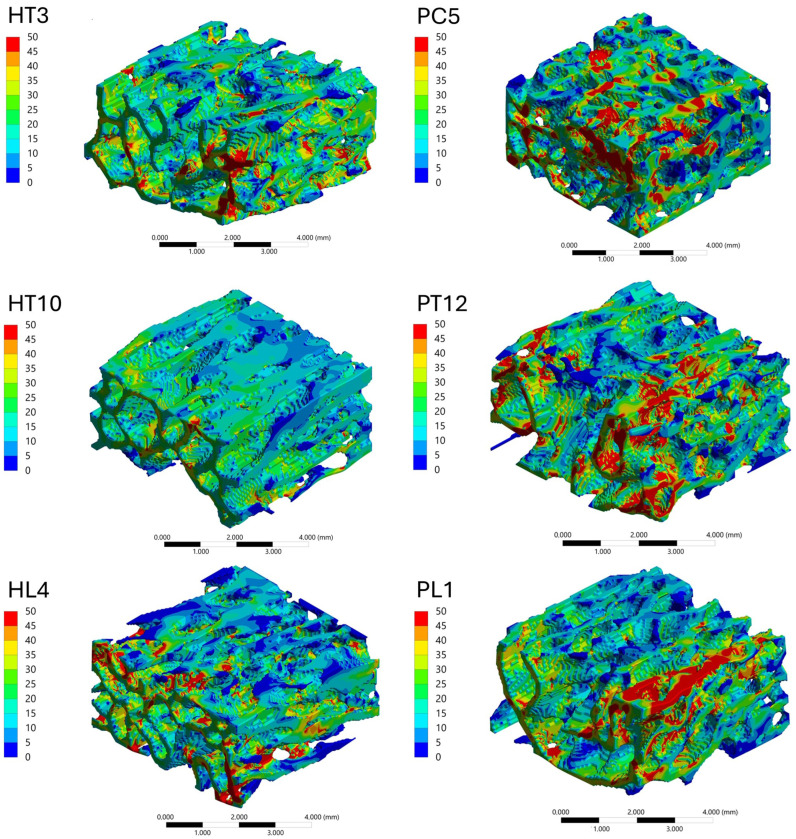
Comparison of the spatial distribution of von Mises equivalent stress (MPa) in vertebral trabecular bone tissue of the Patagonian huemul (*Hippocamelus bisulcus*; left column) and the Southern pudu (*Pudu puda*; right column), displayed using a consistent color scale to enable direct interspecific comparison of stress magnitudes and distribution patterns. Color maps correspond to the cervical (HC5), cranial thoracic (HT3), caudal thoracic (HT10), and lumbar (HL4) vertebrae of the Patagonian huemul, and the cervical (PC3 and PC5), thoracic (PT12), and lumbar (PL1) vertebrae of the Southern pudu. Red tones indicate maximum stress values and blue tones indicate minimum values. The use of a uniform scale across all panels allows direct assessment of differences in stress magnitude and localization between specimens and vertebral regions.

#### 3.4.4. Cranio-Caudal Variation of Mechanical Response

The cranio-caudal variation of the mechanical response was analyzed based on the distribution of von Mises equivalent stress under axial compression (Figure 10). In the Patagonian huemul specimen, the cervical vertebra (HC5) appeared to exhibit multiple localized regions of high stress distributed throughout the trabecular network. HT3 tended to show a more pronounced concentration of stresses, whereas HT10 appeared to display a more uniform stress distribution with lower peak values. The lumbar vertebra (HL4) presented a mixed pattern. A similar cranio-caudal trend was observed in the Southern pudu specimen, with PC3 exhibiting apparently moderate and dispersed stress levels and PC5 presenting more localized stress concentrations.

#### 3.4.5. Interspecific Comparison: Patagonian Huemul vs. Southern Pudu

A direct comparison between the analyzed specimens was performed based on the von Mises stress distributions shown in Figure 10, which displays both species under a uniform color scale and constitutes the primary visual basis for interspecific assessment of stress magnitude and spatial distribution patterns. This addition ensures that the reader is explicitly oriented toward the correct interpretive use of the figure before engaging with the descriptive comparative analysis that follows, without introducing interpretive content beyond what the data support. In general, the Patagonian huemul specimen appeared to exhibit a more distributed stress pattern, with multiple regions of moderate stress distributed throughout the trabecular network. In contrast, the Southern pudu specimen tended to display more pronounced stress localization, with clearly defined high-stress regions concentrated along specific trabecular elements. This pattern was particularly apparent in PC5 and PT12. Overall, both specimens exhibited comparable mean von Mises stress magnitudes under the standardized loading conditions applied, with descriptive differences observed in the spatial distribution and degree of localization of stress concentrations across the trabecular network.

## 4. Discussion

The analysis of vertebral trabecular bone microarchitecture and its mechanical response in the Patagonian huemul and the Southern pudu provides preliminary evidence addressing a key knowledge gap in the axial skeletal biology of native South American cervids [41]. Given the conservation-critical status of both species [28,31] and the documented prevalence of osteopathologies in the Patagonian huemul [26], these findings may contribute to future research with implications for individual management and conservation, while remaining strictly preliminary in scope.

### 4.1. Vertebral Trabecular Bone Microarchitecture

Few studies have addressed the microarchitecture of vertebral trabecular bone in cervids, particularly in native South American species, despite the central role of this structure in locomotion, body support, and protection of the spinal cord [9]. The application of microCT enabled the quantitative characterization of structural parameters in both specimens, providing novel descriptive information relevant for the biomechanical and functional interpretation of their vertebral columns [12,33]. Several studies have demonstrated allometric scaling relationships between body mass and trabecular microstructural parameters [4,5], with evidence that these patterns differ fundamentally between small- and large-bodied mammals and are further modulated by ecology and phylogeny [42,43]. Although the Patagonian huemul, whose body mass is nearly ten times greater than that of the Southern pudu [29,30], showed the expected lower connectivity and bone surface density, it also reduced trabecular thickness, which does not fully align with previously described allometric patterns. An alternative explanation that warrants evaluation in studies with larger sample sizes is that nutritional or physiopathological factors reported in Patagonian huemul populations [26,44] may contribute to these interspecific differences; microCT- and FEA-based studies have demonstrated that micronutrient deficiencies independently reduce BV/TV, Tb.Th, and trabecular number while increasing von Mises stress in vertebral bone [45]; however, such an interpretation cannot be supported by the present specimen-based dataset. Both specimens exhibited a consistent regional pattern, with a progressive increase in trabecular density from the cervical to the lumbar region. In the Patagonian huemul specimen, the cervical region concentrated features of lower trabecular volume, higher porosity, and lower anisotropy, a pattern consistent with a mechanical environment characterized by more variable loading conditions and with the high cervical mobility described in cervids [46,47]; notably, the degree of anisotropy has been identified as the trabecular parameter most closely linked to postural behavior rather than to body mass per se, supporting a functional rather than purely allometric interpretation of this regional contrast [48]. In contrast, the caudal thoracic vertebrae showed greater volume, thickness, and connectivity, lower porosity, and higher anisotropy, consistent with a structurally reinforced configuration associated with the stabilizing role of the thoracolumbar region in quadrupeds [27,49]. In the Southern pudu specimen, trabecular microarchitecture was more uniform along the vertebral column, with trabecular thickness remaining relatively constant across regions, whereas the thoracic and lumbar regions exhibited higher volume, lower porosity, and moderate to high anisotropy, a structural organization consistent with a more homogeneous mechanical response under axial loading [27,49]. Overall, the main interspecific difference was the degree of regional variation: the Patagonian huemul showed a more heterogeneous segmental organization, whereas the Southern pudu exhibited relatively uniform values of thickness, density, connectivity, and porosity, particularly between the thoracic and lumbar regions. This pattern supports the study hypothesis and suggests a more homogeneous distribution of loads along the vertebral column in the Southern pudu, although this interpretation should be considered within the exploratory scope of the study.

### 4.2. Microstructure–Mechanics Relationships

Various experimental studies have demonstrated a close relationship between vertebral trabecular microarchitecture and mechanical response under load. In murine models, BV/TV and Tb.Th are negatively correlated with total deformation and von Mises stress, while Tb.Sp is positively associated with these variables [50]. At the vertebral level, microCT-based finite element studies in human vertebrae have confirmed that BV/TV, SMI, and degree of anisotropy contribute independently to vertebral stiffness [23], and that the combination of bone volume fraction with trabecular microarchitecture and its regional heterogeneity explains a large proportion of the variability in vertebral failure load [24].

Within this framework, the microstructural differences observed between specimens are consistent with the mechanical response patterns recorded in this study. In the Patagonian huemul specimen, HT10 combined high BV/TV and Tb.Th values with the lowest stress and deformation magnitudes, while the remaining vertebrae exhibited a relatively less dense microarchitecture associated with higher mechanical variables. In the Southern pudu specimen, PC5 exhibited lower BV/TV, lower Tb.Th, and higher Tb.Sp, coinciding with elevated stress and deformation values. PC3 presented the lowest stress and deformation values despite lower trabecular density compared to PT12 and PL1, a pattern that may reflect its higher Tb.Th and lower Tb.Sp, highlighting the relevance of interactions among multiple microstructural parameters. These observations are consistent with the notion that trabecular networks with higher density, thickness, and connectivity tend to exhibit greater relative structural stiffness under axial compression [49].

Regarding anisotropy, lower values in cervical vertebrae were consistent with a less directional trabecular organization and may be associated with higher stress levels under axial compression [49]. Greater anisotropy in thoracic and lumbar vertebrae suggests a preferential trabecular orientation favoring more efficient stress distribution [27]. However, this relationship was not consistent across all vertebrae: PC3 showed low anisotropy yet low stress, while HT3 showed high anisotropy yet elevated stress. Therefore, the role of anisotropy in modulating the mechanical response of vertebral trabecular bone remains inconclusive within the present dataset.

### 4.3. Interpretation of FEA-Derived Stress Pattern and Biomechanical Implications

#### 4.3.1. Methodological Consistency and Reliability of Comparisons

A key strength of this study lies in the methodological consistency applied across all analyzed geometries. Methodological consistency across all geometries—uniform voxel size, matched mesh resolution, and standardized meshing criteria—minimizes numerical bias and ensures that differences in mechanical response reflect variations in trabecular architecture rather than modeling inconsistencies [51,52]. The use of mean von Mises stress as the primary metric further reduces sensitivity to local numerical artifacts. This approach enables a robust interspecific comparison, where observed differences in mechanical response can be attributed primarily to variations in trabecular architecture rather than to inconsistencies in numerical modeling [51]. Overall, the methodological framework provides a coherent basis for comparing trabecular mechanical behavior across different anatomical regions and species, strengthening the validity of the conclusions drawn in this study.

#### 4.3.2. Mechanical Interpretation of Trabecular Load Transfer

The analysis of von Mises stress distributions reveals that load transfer within the trabecular bone is governed by a complex network of interconnected structural pathways rather than by homogeneous stress fields. The presence of localized high-stress regions indicates that a subset of trabeculae acts as primary load-bearing elements, forming preferential load paths that channel the applied compressive forces through the structure [52]. In regions where stress is more uniformly distributed, the trabecular network exhibits a more efficient load-sharing mechanism, suggesting a higher degree of structural redundancy. Conversely, highly localized stress concentrations reflect a reduced number of dominant load-bearing trabeculae, which may increase susceptibility to local failure [51]. This distinction highlights the dual mechanical behavior of trabecular bone, where both distributed and localized load transfer mechanisms coexist depending on the underlying microarchitecture. These observations are consistent with the concept of trabecular bone as a cellular solid, where mechanical performance is strongly influenced by connectivity, orientation, and spatial organization of the trabecular network [51,52].

#### 4.3.3. Cranio-Caudal Mechanical Adaptation

The observed variations in stress distribution along the cranio-caudal axis indicate that trabecular bone architecture is adapted to region-specific mechanical demands [53]. Cervical vertebrae tend to exhibit more heterogeneous stress patterns with multiple localized load paths, suggesting a structural configuration capable of accommodating multidirectional loading conditions [53]. In contrast, thoracic and lumbar regions display differences in the degree of stress localization and distribution, reflecting changes in load magnitude and transmission pathways along the spine. In particular, regions with more homogeneous stress fields appear to promote a more effective redistribution of applied loads, reducing peak stress concentrations and potentially enhancing structural durability [53,54]. These findings support the hypothesis that trabecular architecture balances stiffness and load distribution efficiency according to the functional role of each vertebral segment, indicating that cranio-caudal variation is both anatomical and mechanical.

#### 4.3.4. Interspecimen Biomechanical Differences

A clear distinction emerges between the trabecular mechanical responses of the analyzed specimens. The Patagonian huemul specimen generally exhibited a more distributed stress pattern, characterized by multiple load paths and moderate stress levels across the trabecular network. This behavior may suggests a structurally redundant system, where loads are shared among numerous trabeculae, potentially enhancing resistance to localized failure [55,56]. In contrast, the Southern pudu specimen showed more pronounced stress localization, with higher stress concentrations confined to specific regions of the trabecular structure. This may indicates that load transfer is governed by a smaller number of dominant trabeculae, resulting in a less homogeneous stress distribution [56]. While such a configuration may be efficient under certain loading conditions, it may also increase mechanical vulnerability due to the concentration of stresses [57]. From a biomechanical perspective, these differences may reflect specimen-specific structural organization associated with body size, locomotion, and loading environments [23,58]. The Patagonian huemul specimen’s more distributed load transfer mechanism may suggests a structural strategy oriented toward robustness and redundancy, whereas the Southern pudu specimen’s localized stress patterns may indicate a more specialized structural response [55,56]. Importantly, the use of a consistent stress scale across all cases confirms that these differences are not only qualitative but also quantitatively significant [23].

### 4.4. Biological Implications

The following interpretations are inherently speculative, given that the analysis was conducted on a single specimen per species, and should be regarded as hypothesis-generating observations rather than evidence-based conclusions regarding ecological adaptation or habitat preference. The regional patterns of vertebral trabecular microarchitecture observed in the Patagonian huemul specimen provide an anatomical–functional framework that is relevant to the ongoing debate regarding its natural habitat [36,37]. The plasticity of trabecular bone architecture to reflect habitual loading regimes has been extensively documented, and its variation has been used to infer joint loading and locomotor behavior in both extant and fossil taxa [25]. The lower anisotropy and higher relative deformation observed in the cervical segment are consistent with functional descriptions attributing to this region exposure to multidirectional loads and high mobility associated with head and neck movements [47]. In contrast, the denser and more anisotropic trabecular organization of the thoracic and lumbar segments is consistent with a stabilizing role and with axial load transmission toward the caudal region [7,27,49]. These structural features are functionally compatible with locomotor and postural demands associated with topographically heterogeneous environments, including irregular substrates and variable slopes [7,59]. Within this context, the observed pattern is consistent, although not conclusive, with ecologically complex scenarios under the assumption that trabecular microarchitecture reflects habitual loading regimes [25]. However, these interpretations must be considered within the limitations of the study, as the analysis was conducted on a single specimen, which precludes population-level generalizations or direct inferences regarding habitat preference. Despite this limitation, the results provide a biomechanically coherent and internally consistent line of evidence that contributes to evaluating the structural plausibility of different ecological scenarios. Furthermore, they highlight the value of integrative anatomical approaches (microCT–FEA) for supporting conservation-oriented hypotheses in threatened species with limited data availability.

### 4.5. Limitations and Future Directions

The limited number of available specimens (one individual per species) precludes population-level extrapolation and restricts the assessment of intraspecific variability [26]. Additionally, the specimens differ in sex (male huemul vs. female pudu) and age (2 vs. 3 years), which represent potential confounding factors in the interspecific comparison. Sexual and ontogenetic variation in trabecular bone microarchitecture has been described in mammals, and these factors could not be controlled in the present study. Specimen origin and preservation may also introduce variability. The huemul skeleton was obtained through exhumation, whereas the pudu specimen derived from an osteological collection. Although microCT studies suggest that overall trabecular architecture is largely preserved in dry bone [32], potential taphonomic or preservation-related effects on trabecular geometry cannot be fully excluded. From an image-processing perspective, global threshold-based segmentation introduces an inherent source of bias, as threshold selection affects the identification of bone tissue and trabecular geometry [34]. In addition, the removal of unconnected trabeculae during model preprocessing, although necessary for numerical stability, may have slightly reduced the trabecular network and led to minor underestimation of local stress concentrations. The finite element models assumed linear elastic, homogeneous, and isotropic behavior—a simplification widely used in comparative studies [60] that does not fully reproduce the anisotropic and viscoelastic nature of trabecular bone. The single standardized axial load does not reflect multidirectional *in vivo* loading nor the influence of adjacent structures such as intervertebral discs, ligaments, and musculature. Nevertheless, these limitations were partially mitigated through the adoption of homogeneous acquisition, segmentation, and mechanical simulation criteria, allowing the generation of a coherent and reproducible quantitative baseline. Future studies should increase sample size and incorporate ontogenetic, sexual, and geographic variability, as well as develop more advanced computational models integrating complex loading conditions to further elucidate the relationship between trabecular microarchitecture, vertebral biomechanics, and their ecological and pathological implications in South American cervids. In this context, a methodologically relevant direction concerns the experimental validation of the finite element models generated in this study. Since the present models rely on homogeneous and isotropic material assumptions, their mechanical predictions have not been contrasted against empirical surface strain data—a recognized limitation in microCT-based finite element studies of bone [61,62]. Digital speckle pattern interferometry (DSPI) constitutes a technically appropriate approach to address this gap. DSPI is a non-contact, full-field optical technique that enables surface displacement and strain measurements at high spatial resolution across complex specimen geometries, without requiring physical contact or surface modifications that could compromise specimen integrity [61,63]. Unlike conventional strain gauges, which yield only point-specific measurements and provide limited information on spatial strain gradients, DSPI captures continuous strain fields across the entire analyzed surface, thereby offering a substantially more comprehensive basis for finite element model validation [62]. This technique has been successfully applied to validate finite element models of skeletal structures under controlled loading conditions, demonstrating reliable correspondence between experimentally measured and numerically predicted strain distributions in bone specimens of varying complexity [61,63]. The integration of DSPI into future studies on vertebral trabecular bone in South American cervids would thus provide experimental evidence to evaluate the accuracy of the mechanical predictions generated here, refine the constitutive assumptions of the models, and strengthen the biological interpretability of the regional and interspecific differences in mechanical response described in this study.

## 5. Conclusions

This study addressed the relationship between vertebral trabecular bone microarchitecture and its mechanical response under standardized axial loading conditions in two native South American cervids, the Patagonian huemul and the Southern pudu. The integration of microCT-derived structural parameters and finite element analysis provided preliminary support for the working hypothesis, indicating that the mechanical behavior of trabecular bone is closely associated with its underlying microarchitecture. Both specimens exhibited a consistent cranio–caudal gradient in trabecular organization, with lower density, higher porosity, and reduced anisotropy in the cervical region, transitioning toward a denser, more connected, and directionally organized structure in the thoracic and lumbar segments, a pattern associated with regional differences in mechanical response. At the interspecific level, the Patagonian huemul specimen exhibited a more heterogeneous mechanical response, with greater stress localization and higher relative deformation values, particularly in the cervical region. In contrast, the Southern pudu specimen showed a more uniform stress distribution and a more consistent structural response along the vertebral column. These differences were not fully explained by body mass alone, but were associated with variations in trabecular organization, suggesting that specimen-specific microarchitectural configurations may influence mechanical performance under compressive loading. From a broader perspective, this study provides a preliminary comparative baseline of vertebral trabecular microarchitecture and mechanical response in these two cervid species, contributing to addressing a significant knowledge gap in axial skeletal morphology in native ungulates of South America. The non-destructive integration of microCT and finite element modeling represents a promising methodological framework for future studies aimed at exploring the links between bone structure, mechanical function, and ecological or pathological conditions. Ultimately, these findings contribute to advancing the understanding of vertebral functional morphology and may inform future research in conservation biology, particularly in species affected by environmental stressors and population decline, as well as supporting the handling, management, and preservation of biological specimens in both research and conservation contexts. It should be noted, however, that all observations are based on single specimens per species and cannot be extrapolated to population-level conclusions, reinforcing the preliminary and exploratory scope of this study.

## Figures and Tables

**Table 1:** Identification of the vertebral columns analyzed from the Patagonian huemul (*Hippocamelus bisulcus*) and the Southern pudu (*Pudu puda*), including specimen sex, body mass, age, origin, collection date, and recovery site.

Species	Sex	Body Mass	Age	Origin	Collection Date	Recovery Site
*Hippocamelus bisulcus*	Male	75 kg	2 years	Exhumation (2019; Resolution No. 1490, SAG)	20 October 2014	Fauna Andina, Pedregoso Sector, Villarrica; 39°16′ S, 72°13′ W; La Araucanía, Chile; 227 m a.s.l.
*Pudu puda*	Female	8.5 kg	3 years	Donation (Veterinary Anatomy Unit, USS)	2014	N/A

N/A: Not available; USS: Universidad San Sebastián; SAG: Servicio Agrícola y Ganadero (Chilean Agricultural and Livestock Service).

**Table 2:** Microstructural parameters of vertebral trabecular bone tissue in the cervical (HC5), thoracic (HT3 and HT10) and lumbar (HL4) vertebrae of the Patagonian huemul (mean ± standard deviation for Tb.Th and Tb.Sp).

Variable	HC5	HT3	HT10	HL4
BV (mm^3^)	45.11	53.21	70.37	54.23
BV/TV (%)	22.73	26.82	35.47	27.33
BS (mm^2^)	643.47	684.33	680.32	736.82
BS/BV (1/mm)	14.27	12.86	9.67	13.59
BS/TV (1/mm)	3.24	3.45	3.43	3.71
Tb.Pf (1/mm)	3.43	1.29	−0.19	1.41
SMI	1.44	0.60	−0.12	0.62
Tb.Th (mm)	0.25 ± 0.09	0.24 ± 0.08	0.30 ± 0.09	0.23 ± 0.06
Tb.N (1/mm)	0.90	1.11	1.19	1.21
Tb.Sp (mm)	0.66 ± 0.24	0.64 ± 0.22	0.67 ± 0.30	0.58 ± 0.20
FD	2.61	2.62	2.63	2.65
Po(tot) (%)	77.27	73.18	64.53	72.67
DA	0.30	0.76	0.80	0.75

BV: bone volume; BV/TV: bone volume fraction; BS: bone surface; BS/BV: specific bone surface; BS/TV: bone surface density; Tb.Pf: trabecular pattern factor, an index of trabecular connectivity; SMI: structure model index, indicating the prevalence of rod-like versus plate-like trabeculae; Tb.Th: trabecular thickness; Tb.N: trabecular number; Tb.Sp: trabecular separation; FD: fractal dimension reflecting structural complexity; Po(tot): total porosity; DA: degree of anisotropy.

**Table 3:** Microstructural parameters of vertebral trabecular bone tissue in the cervical (PC3 and PC5), thoracic (PT12) and lumbar (PL1) vertebrae of the Southern pudu (mean ± standard deviation for Tb.Th and Tb.Sp).

Variable	PC3	PC5	PT12	PL1
BV (mm^3^)	62.41	56.46	72.35	69.70
BV/TV (%)	31.46	28.46	36.47	35.13
BS (mm^2^)	761.73	741.44	754.67	727.77
BS/BV (1/mm)	12.21	13.13	10.43	10.44
BS/TV (1/mm)	3.84	3.74	3.80	3.67
Tb.Pf (1/mm)	2.07	1.29	−0.54	−0.31
SMI	1.02	0.59	−0.31	−0.18
Tb.Th (mm)	0.30 ± 0.11	0.25 ± 0.10	0.28 ± 0.08	0.28 ± 0.08
Tb.N (1/mm)	1.06	1.13	1.31	1.25
Tb.Sp (mm)	0.58 ± 0.27	0.69 ± 0.34	0.67 ± 0.40	0.66 ± 0.34
FD	2.68	2.65	2.66	2.65
Po(tot) (%)	68.54	71.54	63.53	64.87
DA	0.48	0.35	0.70	0.70

BV: bone volume; BV/TV: bone volume fraction; BS: bone surface; BS/BV: specific bone surface; BS/TV: bone surface density; Tb.Pf: trabecular pattern factor, an index of trabecular connectivity; SMI: structure model index, indicating the prevalence of rod-like versus plate-like trabeculae; Tb.Th: trabecular thickness; Tb.N: trabecular number; Tb.Sp: trabecular separation; FD: fractal dimension reflecting structural complexity; Po(tot): total porosity; DA: degree of anisotropy.

**Table 4:** Total deformation (mm) and von Mises equivalent stress (MPa) obtained from axial compression simulations of vertebral trabecular bone tissue in the cervical (HC5), thoracic (HT3 and HT10), and lumbar (HL4) vertebrae of the Patagonian huemul (*Hippocamelus bisulcus*).

	Total Deformation (mm)				Von Mises Equivalent Stress (MPa)			
	HC5	HT3	HT10	HL4	HC5	HT3	HT10	HL4
Max	$3.33\times 10^{-2}$	$5.34\times 10^{-2}$	$9.43\times 10^{-3}$	$7.52\times 10^{-2}$	818.44	731.84	241.84	608.04
Mean	$1.04\times 10^{-2}$	$4.70\times 10^{-3}$	$2.76\times 10^{-3}$	$3.77\times 10^{-3}$	20.53	21.22	15.95	18.18

HC5: Cervical vertebra C5; HT3: Thoracic vertebra T3; HT10: Thoracic vertebra T10; HL4: Lumbar vertebra L4. Values represent single measurements and not population estimates. Maximum von Mises stress values reflect highly localized stress concentrations at trabecular junctions and thin struts; mean stress values are considered the primary comparative metric.

**Table 5:** Total deformation (mm) and von Mises equivalent stress (MPa) obtained from axial compression simulations of vertebral trabecular bone tissue in the cervical (PC3 and PC5), thoracic (PT12), and lumbar (PL1) vertebrae of the Southern pudu (*Pudu puda*).

	Total Deformation (mm)				Von Mises Equivalent Stress (MPa)			
	PC3	PC5	PT12	PL1	PC3	PC5	PT12	PL1
Max	$2.17\times 10^{-2}$	$3.50\times 10^{-2}$	$2.15\times 10^{-2}$	$2.20\times 10^{-2}$	346.05	416.43	447.62	403.39
Mean	$2.80\times 10^{-3}$	$4.49\times 10^{-3}$	$3.12\times 10^{-3}$	$3.00\times 10^{-3}$	14.26	19.22	18.61	16.18

PC3: Cervical vertebra C3; PC5: Cervical vertebra C5; PT12: Thoracic vertebra T12; PL1: Lumbar vertebra L1. Values represent single measurements and not population estimates. Maximum von Mises stress values reflect highly localized stress concentrations at trabecular junctions and thin struts; mean stress values are considered the primary comparative metric.

## Data Availability

The raw data supporting the conclusions of this article will be made available by the authors on request.

## Supplementary Materials

The following supporting information can be downloaded at: https://doi.org/10.5281/zenodo.19961962, accessed on 1 May 2026. Supplementary Material S1: Raw and processed microCT-derived trabecular morphometric outputs (.txt) for each vertebral volume of interest (VOI), including acquisition settings, segmentation thresholds, and full structural descriptors. Supplementary Material S2: Complete dataset of trabecular microarchitectural parameters (Table S1, PDF) used for interspecific and regional comparisons. Supplementary Material S3: Detailed finite element analysis (FEA) configuration, including mesh convergence analysis, boundary conditions, and modeling parameters implemented in ANSYS. Supplementary Material S4: Representative video files of the scanned vertebral specimens and corresponding volumes of interest (VOIs) used for trabecular microarchitectural analysis, illustrating the spatial definition and localization of the analyzed regions within each vertebral body.

## Abbreviations

The following abbreviations are used in this manuscript:

BS	Bone Surface
BS/BV	Bone Surface/Volume ratio
BS/TV	Bone Surface Density
BV	Bone Volume
BV/TV	Bone volume fraction
CTAn	CT Analyser
DA	Degree of Anisotropy
FD	Fractal Dimension
FEA	Finite element analysis
HC5	Trabecular bone of the cervical vertebra (C5) of the Patagonian huemul
HL4	Trabecular bone of the lumbar vertebra (L4) of the Patagonian huemul
HT3	Trabecular bone of the thoracic vertebra (T3) of the Patagonian huemul
HT10	Trabecular bone of the thoracic vertebra (T10) of the Patagonian huemul
IUCN	International Union for Conservation of Nature
microCT	Micro-computed tomography
mm	millimeter
MPa	Megapascal
PC3	Trabecular bone of the cervical vertebra (C3) of the Southern pudu
PC5	Trabecular bone of the cervical vertebra (C5) of the Southern pudu
PL1	Trabecular bone of the lumbar vertebra (L1) of the Southern pudu
Po(tot)	Total porosity
PT12	Trabecular bone of the thoracic vertebra (T12) of the Southern pudu
SMI	Structure Model Index
STL	Standard Tessellation Language
Tb.N	Trabecular Number
Tb.Pf	Trabecular Pattern Factor
Tb.Sp	Trabecular Separation
Tb.Th	Trabecular Thickness
VOI	Volume of Interest
